# Does import trade liberalization worsen residents’ health? Evidence from China

**DOI:** 10.3389/fpubh.2024.1470338

**Published:** 2024-10-28

**Authors:** Ming Chen, Hongbo Wang

**Affiliations:** ^1^School of Economics and Management, Shanxi University (SXU), Taiyuan, China; ^2^School of Economics, Nankai University (NKU), Tianjin, China

**Keywords:** import trade liberalization, health effect, environmental pollution, public service, income gap

## Abstract

Health is important to human capital and national sustainable development. Based on the latest Chinese Family Panel Studies (CFPS), this paper uses the IVprobit model to test the impact of import trade liberalization on residents’ health from the micro-individual perspective and further explores its mechanism. The results indicate that import trade liberalization brings hidden health costs, and that the negative effects on the health of people in eastern China or rural areas, people with a low education background, people under 60 years of age, and women are more significant. In areas with more serious environmental pollution, the health deterioration effect of expanding imports is more obvious. Increasing green coverage and optimizing the quality of public services can effectively alleviate this negative impact. Import trade liberalization does not affect residents’ health through income gap, therefore when carrying out import trade liberalization the government should pay greater attention to the health status of the specified groups, adopt various means to improve the ecological and living environments, reduce pollution emissions, optimize the supply of public services, and ultimately improve residents’ overall health level.

## 1 Introduction

Health is an unremitting pursuit of human beings and a fundamental condition for social progress. The COVID-19 epidemic that swept the world has set off an unprecedented health crisis worldwide, which has led to a deeper consideration in terms of health problems. In fact, before the outbreak of the epidemic, China’s health crisis caused by environmental pollution had reached a critical juncture. Water pollution incidents, such as the cyanobacteria pollution in Taihu Lake and the Songhua River, are a frequent occurrence in China, which not only destroys the balance of the surrounding ecological environment, but also threatens normal social operations. The increasingly severe haze weather has increased the risk to people suffering from respiratory, cardio-cerebrovascular, cancer, and infectious diseases. China’s health and safety issues have attracted great attention from the government and the country’s citizens. From the first National Health Conference in the new century to the Healthy China 2030 Plan issued by the State Council in 2016, to the proposal of the Healthy China development strategy at the 19th National Congress of the Communist Party of China in 2017, China’s actions to promote people’s health are advancing steadily. China’s health undertakings have made great progress, and the health status of residents has been greatly improved. The average life expectancy in China has risen from 35 years at the beginning of the founding of the People’s Republic of China in 1949 to 78.6 years in 2024. Neonatal mortality and maternal mortality have also achieved the United Nations Millennium Development goals ahead of schedule. The proportion of older adults people aged 65 and above receiving health management in primary healthcare institutions nationwide has reached 62.5%, and the level of residents’ health literacy has increased from 17% in 2018 to 29.7% in 2023. The overall level of people’s health has reached that of the average of middle- and high-income countries, and the high performance in health achieved by the government has created huge health dividends. However, with the acceleration of industrialization, urbanization, and population aging, the road to national health in China still faces many challenges. The healthy population in China accounts for only 5% of the total population, while those diagnosed with various diseases account for 20%. About 75% of the population is in a sub-healthy state. Psychological health problems manifested in stress, anxiety, and depression are also widespread in modern urban populations. While accelerating high-quality economic development and firmly stepping into a moderately prosperous society, the health of the entire population—which is related to people’s livelihood—has become a new issue for the government.

Currently, the world as a whole is undergoing major changes unseen in a century, and China as an individual country is also experiencing a critical period of transformation and upgrading. Based on the comprehensive consideration of the internal demand for economic development and the construction of an open world economy, the Chinese government has formulated a long-term strategy of opening up to the outside world. Since 2010, China has successively introduced a number of tariff measures to reduce the import duty of various commodities, covering a wide range of goods, including mechanical and electrical equipment, textiles and building materials, resource commodities and primary processing products, equipment and parts needed by strategic emerging industries, disaster relief supplies and other industries. The implementation of import trade liberalization has brought a variety of external consumer goods to Chinese residents, which helps to improve domestic consumption. At the same time, the import of scarce resources and technology-intensive products such as agricultural, energy, and high-tech goods has promoted the effective connection of production, distribution, circulation, and consumption, and played an important role in promoting domestic technological innovation and forcing industrial transformation. Generally speaking, the complex impact of import trade liberalization has penetrated all aspects of Chinese production and life, and the evaluation of the welfare effect of expanding imports should also be based on a comprehensive perspective. It is thus necessary to study the health effect of import trade liberalization.

Because most countries tend to adopt an export-oriented strategy to build the necessary economic base, a few early foreign studies have discussed the impact on health from the perspectives of export and trade opening (1). This has gradually turned into research on the relationship between import trade liberalization and health. Colantone et al. (2), Giuntella et al. (3) and Atkin (4) took mental distress, obesity rate, and nutritional intake, respectively, as proxy variables for health, and came to the conclusion that import trade liberalization in Britain, Mexico, and India was not conducive to the health of local residents. With the continuous expansion of the United States’ trade deficit, studies on the health effect of import trade liberalization based on the US have also emerged in recent years. However, they mainly focused on the impact of China’s import trade on the health of US residents. Most studies measured the health status of residents with injury rate, mental health, self-rated health, mortality, and other indicators. They agreed that the import competition and trade shock from China would aggravate the health risks of US residents (5, 6). Relatively few works focus on China, and these are mainly from the perspective of export expansion (7, 8). However, the literature on the health effect of trade liberalization from the perspective of import expansion is quite lacking, and only three related studies have been found to date. Fan et al. (9) studied the impact of import tariff reduction on the health of Chinese manufacturing workers as a whole and on workers with different skill levels. The focus of this paper is mainly on manufacturing workers. It found that the reduction of import tariffs will increase the working hours, which is not conducive to the health of workers, and widen the income, health gap and welfare gaps between skilled and unskilled workers. Zhang (10) used the data of CHNS to explore the residents’ health effect of import trade liberalization since China’s accession to the WTO (1997–2009) and believed that import shock had a negative impact on residents’ health through the adjustment of the labor market and diet structure. Lei et al. (11) confirmed that import expansion is conducive to improving residents’ health through the study of relatively macro provincial-level import tariff rates and CGSS data.

With the successive promulgation of China’s policies on lowering import tariffs in recent years, it is necessary for us to explore the complex relationship between the two based on the latest trade and health data that are closest to reality. This paper takes the trade and residents’ health status after 2010 as the background, uses the Chinese Family Panel Studies (CFPS) conducted by the China Institute of Social Science Survey of Peking University to make an in-depth study of the overall impact of import trade liberalization on residents’ health from 2010 to 2018.^1^ The reliability of the conclusions is verified through various robustness methods. Meanwhile, the heterogeneity of this impact is explored based on different criteria such as region, education, age, gender, and urban–rural status. In addition, this article examines the mechanism of imports on residents’ health from three aspects: environment; public services; and income inequality. Compared with existing literature, the innovation of this paper lies mainly in the following three aspects: (1) Health is the foundation of the life of the individual and the nation, and effects should be taken seriously when evaluating any trade policy. However, in the context of China, there is still limited specialized literature on the relationship between import trade liberalization and health, and some of these studies mainly focus on the impact on manufacturing workers, while research on the impact on the health of all adults is relatively rare. Therefore, this study provides an effective supplementary analysis of the welfare effects of import trade liberalization from a broader perspective, enriching the theoretical research in this field and greatly promoting interdisciplinary research. (2) This paper uses a series of subjective and objective indicators, such as self-rated health, memory status, incidence rate and mental health, to avoid the problem of using self-rated health alone, which may make the results too subjective. Then, through the use of the empirical research methods of Probit, IVprobit and LPM, we more accurately grasp the multiple effects of import expansion on health. (3) The manuscript further refines the depth of such research from the provincial level to the prefecture city level and explores the impact channels of import trade liberalization on residents’ health from multiple perspectives and using multiple indicators, providing a solid theoretical basis and rich policy insights for decision-makers to implement macroeconomic regulation that is more conducive to people’s health.

## 2 Mechanisms for import trade liberalization’s effects on health

Combined with the Grossman (12) health demand model, we believe that trade liberalization may affect the health of residents in the following three ways: environmental conditions; basic public services; and income inequality.

Academic circles have essentially reached a consensus on the negative effects of environmental pollution on residents’ health (13). However, there remain differences in the relationship between trade liberalization and environmental conditions. On the one hand, some scholars believe that, due to the technical effect of import trade, trade liberalization does not lead to an increase in pollution emissions. For example, Lovely and Pop (14) found that technology exchange and knowledge spillover accompanied by free trade could make some areas more likely to obtain cleaner production technology, which is conductive to pollution prevention and control and improves residents’ health. Guo (15) used micro data from 2011 to 2012 and found that the import of intermediate goods would reduce enterprises’ pollution emissions through the resource allocation and technology spillover effects. On the other hand, some studies insist on the view that trade openness leads to an increase in pollution emissions, which is harmful to the health of residents. For example, Managi et al. (16) thought that trade liberalization might enable underdeveloped countries to attract pollution-intensive products of transnational corporations through loose environmental standards, thus increasing the emissions of sulfur dioxide and carbon dioxide in non-OECD countries. Cole (17) found that trade liberalization promoted the migration of polluting industries from developed countries to developing countries, and then accelerated the depreciation rate of residents’ health, which verifies the pollution paradise hypothesis. In addition, some other studies believe that imports will expand the production scale of enterprises, leading to an increase in total pollution emissions, while also reducing the emission intensity of companies due to the increase in emission reduction investment (18).

A perfect public education service can lead to a wider range of citizens having good cultural literacy, and a complete health and medical service system would also make the prevention and treatment of diseases more convenient for residents. The positive impact of sound public services on health has been verified by a large number of studies (19–21). Most investigations support the positive promotion relationship between import trade liberalization and public services from the perspectives of foreign trade risk and government tax. The former mainly believes that trade liberalization exposes a country to a large number of external shocks. Governments of low-income and high-income countries currently increase public expenditures to improve the life quality of residents and reduce trade risks (22). The latter posits that the import expansion caused by tariff reduction may increase government revenue in two ways and thus improve the quality of public services. First, the decline in the price of imported intermediate goods reduces the production costs of domestic manufacturers and expands the output scale, thus increasing the tax base and tax revenue. Second, the price preference of intermediate products is transmitted to the final products layer by layer, which enhances competitive advantage in the final product export price, and the tariff revenue increases with the expansion of export scale (23). The assumption that the increase in fiscal revenue will promote the government to provide more public services has also been confirmed by other relevant Chinese literature (24).

When exploring the role of income inequality in the health effect of import trade, we divide the analysis into two stages: import trade liberalization on income disparity; and income disparity on health. In the first stage, some studies believe that, compared with high-skilled workers, import trade liberalization will significantly reduce the employment of low-skilled workers, thus increasing income inequality (25). Other studies have found that import trade liberalization can promote rural or low-skilled workers engaging in informal or non-agricultural employment, thus narrowing the income gap (26). Therefore, the specific impact of expanding imports on income inequality is not uniform. In the second stage, the impact of the income gap on health can also be divided into two categories. Most studies have found that the widening of the income gap damages health to some extent (27–29). The influence mechanism includes (1) the lack of social trust increases more factors leading to social unrest, such as conflicts and crimes (30); (2) it increases the negative emotions and psychological pressure for individuals, which leads to stress-induced behavior and aggravates health deterioration (31); (3) the stratified expansion of public service demand among high- and low-income groups makes it easy for the government to underestimate the value of public goods, which makes it difficult for low-income people to enjoy the public services that they deserve (32); (4) it increases the credit constraints of low-income groups to reduce the long-term benefits of medical investment (33). On the other hand, a few studies have found that income inequality is beneficial to residents’ health (34), mainly because the demand of high-income people for a higher level of public services stimulates technological innovation, and the resulting technology spillovers bring about external positive effects on the health improvement of other groups. At the same time, under the progressive tax system, the widening income gap increases the government’s tax revenue to a certain extent, setting a more solid foundation for the government to increase public resources expenditure, and ultimately increasing residents’ access to health resources (35). The reason why so many studies come to opposing conclusions is, we believe, mainly related to the selection of research objects, data, health measurement indicators, and empirical methods.

From the above mechanism analysis, we can draw two basic hypotheses:

*H1*: In general, import trade liberalization will have an impact on the health of residents, but the direction is uncertain.

*H2*: Environmental conditions, public service quality, and income disparity may be potential channels for trade liberalization to affect residents’ health.

## 3 Data and methodology

### 3.1 Individual health

The data used in this paper to measure individual health are from the CFPS conducted by the China Institute of Social Science Survey of Peking University. The CFPS is a national, large-scale social tracking survey project started in 2010. The survey covers 25 provinces, cities, and autonomous regions in China. At present, the tracking survey data from 2010, 2012, 2014, 2016, and 2018 have been published. This paper mainly sorts out the adult data of the CFPS^2^ and deletes the samples with “refuse to answer,” “not applicable,” “unclear,” and abnormal values in the options. Finally, data from 47,805 samples from 114 cities in 24 provinces are obtained. There are 50 cities in the east and 64 cities in the central and western regions.^3^ The regional distribution is reasonable and representative.

The question about residents’ health in the CFPS survey project is “What do you think of your health status?” The options are assigned values of 1–5, corresponding to “healthy,” “average,” “relatively unhealthy,” “unhealthy,” and “very unhealthy,” respectively, in 2010, corresponding to “very healthy,” “healthy,” “relatively healthy,” “average,” and “unhealthy” in 2012–2018. That is to say, the options corresponding to this question may vary in different years. As a result, it is difficult to compare the health of individuals across periods. In order to make them comparable in different periods, we divide the above options into binary values. When selecting “healthy,” “very healthy,” and “relatively healthy” in the above options, we think that the residents are in a “healthy” state. When selecting “unhealthy” they are considered to be in an “unhealthy” state. This distinction is relatively easy to make. Because the health information represented by the option “average” is vague, for the sake of safety we treat the “average” option as “unhealthy” and “healthy,” respectively, when we divide the ordered health data. Finally, the residents’ health data under the two classification methods are obtained. Selfhealth1 and selfhealth2 are used to denote the classification when “average” is treated as “unhealthy” and “healthy,” respectively. If the residents are healthy, this is expressed as 1, and as 0 otherwise. Self-rated health can comprehensively measure the physiological health status of individuals and is the most representative health index. The binary classification of “healthy” and “unhealthy” based on subjective self-assessment has been a common practice in academic research on health issues, which has been confirmed by many high-quality studies (36–38).

In addition to the self-rated health, other questions in CFPS questionnaires can also reflect the physical condition of the interviewees. Specifically, the question regarding residents’ sickness in CFPS is “Have you been unwell in the past 2 weeks?” and the options “yes” and “no” correspond to the values 1 and 0. The question “Can you remember the main things that happened to you in the past week” reflects residents’ memory; the options are “completely able to remember,” “able to remember the majority,” “able to remember half,” “only a few things,” and “only a little bit.” Like self-rated health, the information represented by the middle option, “able to remember half,” is also ambiguous and can indicate either good or bad memory. When the value of memory1 is 1, it means “completely able to remember,” “able to remember the majority,” “able to remember half,” and indicates that the residents have better memory, otherwise the value is 0; when the value of memory2 is 1, it means “completely able to remember,” “able to remember the majority,” and shows that the residents’ memory is better, otherwise the value is 0. Generally speaking, being too thin or obese may cause diseases such as osteoporosis, anemia, hypertension, coronary heart disease, and diabetes. We use the internationally accepted body mass index (BMI) to measure the degree of individual health. The formula is BMI = weight (kg)/height^2^ (m^2^); in the samples, a BMI of between 18 and 28 is set to 1, and a BMI less than 18 or greater than or equal to 28 is set to 0. In this paper, the above health information is considered a substitute variable for selfhealth1 and included in our analysis.

Mental health is another important aspect for measuring an individual’s overall health. The CFPS investigated the psychological state of interviewees with the question “How often did you experience the following mental feelings or behaviors in the last week/month?” With regard to this issue, the options provided in the survey questionnaires from different years vary and include two types of statistical cycles: weekly or monthly. For this reason, we select three similar options in different years that can reflect the mental health status of individuals and correspond to the three descriptive options “I cannot be excited about anything,” “It is difficult to do anything,” and “I think life is meaningless” in 2010 and 2014, and “I feel depressed,” “I find it hard to do anything,” and “I do not think life can continue” in 2012, 2016, and 2018. The options “never,” “sometimes,” “half of the time,” and “often and almost every day” under the weekly statistical period correspond to “almost none,” “sometimes,” “often,” and “most of the time” under the monthly statistical period and are assigned the values 0–3. Next, the three scores are averaged, and the final value of the group with a score greater than the median is set to 1, which represents the part of the population with a relatively poor mental state, otherwise it is 0.

### 3.2 Measurement of import trade liberalization

Based on the practice of Dai et al. (25), this paper uses the import tariff rate to measure the level of regional import trade liberalization. The specific measurement formula is as follows:


(1) $${\mathrm{tariff}}_{ct}=\underset{j}{\sum }\eta_{jc}{\mathrm{Outputtariff}}_{jt}$$

(2) $${\mathrm{Outputtariff}}_{jt}=\frac{\sum_{s\in K_{j}}{num}_{st}\times {\mathrm{tariff}}_{st}^{HS6}}{\sum_{s\in K_{j}}{num}_{st}}$$

(3) $$\eta_{jc}=\underset{j}{\sum }\frac{{p_{jc}}_{,2010}}{\sum_{j}p_{jc,2010}}$$

In Equations 1–3, ${\mathrm{Outputtariff}}_{jt}$ denotes the import tariff rate of the final products, of the industry, j represents the industry, c represents the city, and $K_{j}$ represents the product set of industry j; ${num}_{st}$ and ${\mathrm{tariff}}_{st}^{HS6}$ denote the number of tax items and import tariff rate of six-digit code product s in year t, respectively. With the help of the conversion table between HS2007–HS2002, HS2012–HS2002, and HS2017–HS2002, the tariff data are unified into the HS2002 version. The conversion relationship between HS2002 and the International Standard Industrial Classification [ISIC(Rev3)] and the conversion relationship between the Industry Classification of National Economy in 2002 (GB/T2002) and the International Standard Industry Classification [ISIC(Rev3)] are used to match HS2002 with GB/T2002, and then the six-digit code import tariff data is integrated into the industry tariff data. $\eta_{jc}$ is the employment weight when the industry-level tariff is weighted average. $p_{jc}$ is the number of the city-industry labor force—that is, the labor force share of an industry in the total urban labor force in the initial year (2010). The import tariff data are from the WTO’s Tariff Download Facility database, and the labor force data are from China’s industrial enterprise database.

### 3.3 Control variables

There are many factors that affect individual health. Having referred to previous studies on health-influencing factors, this paper adds control variables from the individual, family, and macro levels to reduce the estimation bias caused by missing variables. The individual level mainly includes age, gender, work status, marital status, medical insurance, smoking, drinking, household status, education level, and so on. The family level includes three variables: family population; per capita household net income; and domestic water quality. The number of people living with the interviewee and the economic status of the family largely determine the amount of living resources allocated to each individual, and family structure may also indirectly affect individual health by influencing family lifestyle. Water is the source of life and a necessary substance for normal metabolism in the human body. The safety of domestic water is closely related to personal health. The macro level includes: (1) the regional economic situation, measured by city per capita GDP; (2) the development of secondary industry, measured by the proportion of the added value of the secondary industry in GDP; (3) the development of tertiary industry, measured by the proportion its added value in GDP; (4) the level of urbanization, expressed by the proportion of non-agricultural employment in the total employment population; (5) the savings rate, measured by the proportion of urban and rural residents’ savings deposit in GDP at the end of the year; and (6) the per capita fiscal expenditure, calculated by the ratio of government budget expenditure to the total population at the end of the year. Human beings are products of society, and the improvement of individual health cannot be achieved without the optimization of the social environment in which they live. The regional economic situation, the level of development of major industries, and the urbanization rate may have an impact on individual income levels and health status by affecting employment rates. The savings rate is not only a barometer of regional economic health, but also a key indicator of individual and household financial health. In addition, fiscal expenditure constitutes the material guarantee for the construction of urban health infrastructure and the allocation of basic medical resources. The control variables at the individual and family levels are derived from the CFPS survey data, the macro level data come from the EPS statistical database and the Wind Financial Terminal.

### 3.4 Model construction

This paper constructs the following econometric model to examine the impact of trade liberalization on individual health:


(4) $${\mathrm{Health}}_{ict}=\beta_{0}+\beta_{1}{\mathrm{tariff}}_{ct}+\lambda_{1}Z_{ict}+\lambda_{2}N_{fct}+\lambda_{3}H_{ct}+\varepsilon_{ict}$$

In Equation 4, i represents an individual resident, c represents the area where the individual is located, t represents year, ${\mathrm{Health}}_{ict}$ represents the health level of the individual, ${\mathrm{tariff}}_{ct}$ represents the level of import trade liberalization, $Z_{ict}$ represents the set of individual control variables, $N_{fct}$ is the set of household control variables, $H_{ct}$ is the set of regional macro control variables, and $\varepsilon_{ict}$ is the random disturbance item. This article selects adult samples from all years disclosed by the CFPS to construct a five-year balanced panel survey data. In this paper, the explained variables are binary, and taking into account that there may be some common factors affecting import tariff and health, we built an IVprobit model to explore the impact of import trade liberalization on residents’ health status and solve the possible problems of endogeneity. Because the tariff level of the year before the investigation period is often a strong predictor of the future tariff reduction rate (25) and is not affected by the health status of the following years, the tariff in 2009 is a more appropriate instrumental variable for regional trade liberalization. The tariff calculation in 2009 is based on Equation 1, changing the weight $\eta_{jc}$ to the labor force proportion in 2009, and then calculating the weighted average of the industry import tariff of the final product in the same year. The descriptive statistics of the main variables are shown in Table 1.

**Table 1:** Descriptive statistics.

Variables	Variable explanation	Mean	SD	Min	Max
selfhealth1	Self-rated healthy1, the option “general” represents unhealthy, healthy = 1, unhealthy = 0	0.601	0.490	0	1
selfhealth2	Self-rated healthy2, the option “general” represents healthy, healthy = 1, unhealthy = 0	0.822	0.383	0	1
ifsick	Did you feel unwell in the past 2 weeks? Yes = 1, No = 0	0.319	0.466	0	1
memory1	Memory1, option “can remember half” means good memory	0.734	0.442	0	1
memory2	Memory2, option “can remember half” means bad memory	0.475	0.499	0	1
BMI	Body Mass Index, when between 18 and 28 is 1, the rest is 0	0.693	0.461	0	1
mood	Mental health	0.524	0.600	0	1
tariff	Import trade liberalization	0.895	0.189	0.360	1.380
age	Age	50.705	13.007	16	94
work	Have a job or not, yes = 1, no = 0	0.683	0.465	0	1
marri	Marital status, married (with spouse), cohabitation = 1, divorce, widowed, unmarried = 0	0.912	0.284	0	1
insurance	Have a medical insurance or not, yes = 1, no = 0	0.922	0.268	0	1
ifsmoke	Did you smoke in the past month? Yes = 1, No = 0	0.301	0.459	0	1
ifwine	Did you drink more than three times a week in the past month? Yes = 1, no = 0	0.167	0.373	0	1
gender	Gender, male = 1, female = 0	0.480	0.500	0	1
hukou	Household status, agricultural household = 1, non-agricultural household = 0	0.707	0.455	0	1
education	The highest education, Kindergarten/illiterate/semi-illiterate = 0, primary school = 6, junior high school = 9, senior high school/technical secondary school/technical school/vocational high school = 12, junior college = 15, undergraduate = 16, master = 19, doctor = 23	6.734	4.741	0	19
familysize	Family size	4.076	1.816	1	26
famperinc	Family per capita net income	13,023.240	17,516.900	0	833,000
water	Domestic water, tap water, mineral water/purified water/filtered water = 1, others = 0	0.661	0.473	0	1
pgdp	Regional per capita GDP	44,173.730	29,403.030	5134.560	174,000
secrate	Proportion of added value of secondary industry in GDP	138.087	75.739	1	290
thirdrate	Proportion of added value of tertiary industry in GDP	155.334	80.603	1	298
urbanization	Urbanization level	65.181	16.283	1	77
savrate	Saving rate	230.134	120.733	1	442
tariffempnin	Tariff level in 2009 measured by employment weight	0.091	0.020	0.041	0.133
tariffoutnin	Tariff level in 2009 measured by weight of gross industrial output value	0.090	0.022	0.034	0.212
penetration	Trade penetration	0.238	0.378	0.001	2.696
green	Green coverage rate	199.900	117.661	1	421
pm2.5	PM2.5	186.632	152.090	1	453
wastewater	Industrial wastewater discharge	261.993	155.758	1	537
gas	Industrial smoke emission	275.772	160.771	1	551
midsch	Number of secondary schools per 10,000 people	156.311	84.190	1	320
agency	Number of health institutions per 10,000 people	282.638	154.368	1	543
technicians	Health technicians per 10,000 people	274.946	174.292	1	569
gaprate	Income gap—ratio	2.530	0.907	1.290	14.525
gapredu	Income gap—difference	13,106.410	4615.229	4489.440	33,086.500

Calculated by the authors.

## 4 Empirical results

### 4.1 Full sample benchmark regression

For comparison, we first use the probit model to examine the impact of trade liberalization on residents’ health. Column (1) of Table 2 shows that the estimated coefficient for tariffs on health is significantly positive at the 5% level when control variables such as regional economic development level, the proportion of secondary and tertiary industries in GDP, urbanization level, and savings rate are added, and the fixed effects of cities and years are controlled—that is, the decline in tariffs will significantly reduce the health level of residents. The regression results of IVprobit are shown in column (2). After dealing with the possible endogenous estimation bias, it still shows that tariff reduction has adverse effects on residents’ health.

**Table 2:** Overall regression and partial robustness test: change in regression method and instrumental variables.

Variables	Probit	IVprobit	LPM	IVprobit -output	IVprobit -penetration
	(1)	(2)	(3)	(4)	(5)
tariff	0.6919**(0.353)	1.1166*(0.5871)	0.2626*(0.1247)	1.1166*(0.5871)	6.6599**(3.1339)
age	−0.0167***(0.0008)	−0.0167***(0.0008)	−0.0058***(0.0003)	−0.0167***(0.0008)	−0.0166***(0.0008)
work	0.1663***(0.0172)	0.1662***(0.0172)	0.0611***(0.0063)	0.1662***(0.0172)	0.1646***(0.0173)
marri	−0.0220(0.0295)	−0.0220(0.0295)	−0.0035(0.0102)	−0.0220(0.0295)	−0.0219(0.0294)
insurance	−0.0086(0.0249)	−0.0086(0.0249)	−0.0024(0.0087)	−0.0086(0.0249)	−0.0082(0.0249)
ifsmoke	0.0483**(0.0230)	0.0483**(0.0230)	0.0170***(0.0079)	0.0483**(0.0230)	0.0475**(0.0229)
ifwine	0.2100***(0.0228)	0.2101***(0.0228)	0.0720***(0.0076)	0.2101***(0.0228)	0.2114***(0.0228)
gender	0.1588***(0.0230)	0.1588***(0.0230)	0.0549***(0.0080)	0.1588***(0.0230)	0.1580***(0.0229)
hukou	−0.0017(0.0258)	−0.0020(0.0258)	0.0012(0.0090)	−0.0020(0.0258)	−0.0051(0.0258)
education	0.0250***(0.0023)	0.0250***(0.0023)	0.0090***(0.0008)	0.0250***(0.0023)	0.0248***(0.0023)
familysize	0.0172***(0.0049)	0.0172***(0.0049)	0.0057***(0.0017)	0.0172***(0.0049)	0.0176***(0.0048)
lnfamperinc	0.0268***(0.0045)	0.0268***(0.0045)	0.0097***(0.0016)	0.0268***(0.0045)	0.0269***(0.0045)
water	0.0737***(0.0192)	0.0735***(0.0192)	0.0250***(0.0068)	0.0735***(0.0192)	0.0697***(0.0193)
lnpergdp	−0.2395***(0.0683)	−0.2477***(0.0735)	−0.0822***(0.0241)	−0.2477***(0.0735)	−0.3544***(0.0903)
secrate	−0.0006(0.0004)	−0.0005(0.0005)	−0.0002(0.0002)	−0.0005(0.0005)	0.0006(0.0008)
thirdrate	−0.0009**(0.0004)	−0.0008**(0.0004)	−0.0003*(0.0001)	−0.0008**(0.0004)	−0.0001(0.0006)
urbanization	0.0021**(0.0010)	0.0021**(0.0010)	0.0007*(0.0004)	0.0021**(0.0010)	0.0029***(0.00110)
savrate	−0.0004***(0.0001)	−0.0004***(0.0001)	−0.0001***(0.0000)	−0.0004***(0.0001)	−0.0005***(0.0001)
Constant	1.9868**(0.7945)	1.6054**(0.6338)	1.3411***(0.2863)	1.6054**(0.6338)	−3.3830(2.9255)
City FE	YES	YES	YES	YES	YES
Year FE	YES	YES	YES	YES	YES
cluster	YES	YES	YES	YES	YES
Obs.	47,805	47,805	47,805	47,805	47,805

Standard errors are given in parentheses; ***p < 0.01, **p < 0.05, *p < 0.1.

The results for the individual and family control variables show that with the increase in age, health status will continue to decline, which accords with the law of human development. Men are healthier than women. The highly educated and the employed are generally healthier than the less educated and the unemployed, perhaps because the former two groups are more aware of the need for maintenance. At the same time, the relatively steady income situation also makes them more flexible with regard to food availability and makes it easier to meet daily food nutrition intake needs. The health status of smokers and drinkers is better than that of non-smokers or non-drinkers. We think that the possible reason for this unexpected result is that the above two questions in the CFPS questionnaire selected in this paper only investigated whether the individual has ever performed the above behaviors and does not involve specific measurement of smoking or drinking. Therefore, the respondent cannot be arbitrarily judged on excessive smoking or drinking. In fact, a small amount of drinking can promote blood circulation, prevent kidney disease, diabetes, and other diseases, and a small amount of smoking may also play a positive role in relieving stress and regulating mood. It is not hard to understand why purchasing medical insurance has no significant impact on health. Although medical insurance can reduce the financial burden of individual medical treatment, allowing people to enjoy more adequate health examinations, along with a wider variety and higher quality health services, thereby positively promoting the level of personal health (39), it is also possible that the decrease in medical expenses may lead to more health risk behaviors (40). Therefore, the impact of medical insurance on residents’ health is not absolute. In addition, marital status and household registration have no significant impact on residents’ health. The greater the family population is, the more likely it is to keep healthy. There may be two reasons for this: the first is that a large family size increases the family members’ attention to daily diet, and the second is that the harmonious family relationship and mutual companionship between members make it easier for individuals to maintain a good attitude and reduce the probability of mental illness or other physiological diseases. The higher the family per capita income level, the better the health status of residents. The quality of domestic water has a direct and positive impact on people’s health.

### 4.2 Robustness test

#### 4.2.1 Change in calculation method

In the case of a large sample, the regression results of linear probability (LPM) and probit models tend to be consistent (41, 42). Therefore, we add a fixed effect to the linear probability model to retest the conclusion of the benchmark regression. According to the estimation result in column (3) of Table 2, the decline of import tariffs will significantly reduce the health level of residents.

#### 4.2.2 Change in calculation method of instrumental variable

To ensure the accuracy of the instrumental variable, we use the weight of the gross industrial output value to recalculate the tariff level of the year before the initial year (2009)—that is, the $\eta_{jc}$ in Equation 1 is changed to the share of the total industrial output value of a certain industry in the total industrial output value of a city. The regression result of Table 2 model (4) shows that the recalculation of the instrumental variable does not change the coefficient and significance of the core independent variables.

#### 4.2.3 Reselect instrumental variable

Trade penetration is an index closely related to import tariffs. The change in the import tax rate changes the import and export trade volume, and then directly affects the proportion of imports and exports in a region’s total consumption. Therefore, we take the penetration rate as the instrumental variable of trade liberalization for regression. The calculation formula for trade penetration rate is: ${\mathrm{penetration}}_{ct}=\frac{{\mathrm{trade}}_{ct}}{{\mathrm{import}}_{ct}+{GDP}_{ct}—{\mathrm{export}}_{ct}}$ . Here ${\mathrm{trade}}_{ct}$ , ${\mathrm{import}}_{ct}$ , ${\mathrm{export}}_{ct}$ , and ${GDP}_{ct}$ are the trade value, import value, export value, and regional GDP of c city in period t. From the regression coefficient and the significance of tariffs in column (5) of Table 2, we can see that trade liberalization will still have a negative impact on residents’ health.

#### 4.2.4 Recalculation of health

Although self-rated health has strong subjectivity, some studies have confirmed that it is highly correlated with objective indicators such as mortality, and it is an effective indicator to reflect health status. At the same time, we fully tap into other information that can reflect the health status of residents in the CFPS project and add other indicators that can reflect the objective health and mental health of residents in the robustness test to realize the scientific and comprehensive measurement of individual health status to the greatest extent. In columns (1)–(5) of Table 3, the indicators of self-rated health2, prevalence, memory1, memory2, and mood are used as substitute indicators for health1 in regression. The conclusion that import trade liberalization will bring health costs does not change. The reason why import expansion increases people’s mental distress may be that import competition increases the work pressure among residents and lowers their expectations for the future (2).

**Table 3:** Robustness test: remeasure explained variables.

Variables	Selfhealth2	Ifsick	Memory1	Memory2	Mood
	(1)	(2)	(3)	(4)	(5)
tariff	1.9220***(0.7174)	−1.7901***(0.5909)	1.0837*(0.6527)	2.3628***(0.6214)	−2.4782***(0.8551)
age	−0.0167***(0.0010)	0.0127***(0.0008)	−0.0106***(0.0008)	−0.0122***(0.0008)	0.0050***(0.0010)
work	0.2533***(0.0208)	−0.0446***(0.0171)	0.0420**(0.0178)	−0.0223(0.0171)	−0.1444***(0.0241)
marri	−0.0254(0.0372)	−0.0685**(0.0283)	0.0719**(0.0284)	0.0065(0.0288)	−0.2361***(0.0340)
insurance	−0.0228(0.0300)	−0.0083(0.0251)	0.1236***(0.0263)	0.1100***(0.0251)	−0.1432***(0.0339)
ifsmoke	0.0647**(0.0290)	0.0323(0.0227)	0.0159(0.0232)	0.0314(0.0222)	0.0719**(0.0309)
ifwine	0.2478***(0.0295)	−0.1462***(0.0231)	0.0639***(0.0231)	0.0708***(0.0220)	−0.0612*(0.0319)
gender	0.1377***(0.0290)	−0.2781***(0.0224)	0.1388***(0.0229)	0.1360***(0.0222)	−0.1787***(0.0311)
hukou	−0.1498***(0.0328)	−0.0306(0.0255)	−0.1530***(0.0269)	−0.1931***(0.0251)	0.0681*(0.0353)
education	0.0355***(0.0029)	−0.0126***(0.0022)	0.0547***(0.0022)	0.0602***(0.0022)	−0.0346***(0.0028)
familysize	0.0316***(0.0059)	−0.0199***(0.0047)	0.0012(0.0048)	−0.0023(0.0046)	−0.0141**(0.0065)
lnfamperinc	0.0188***(0.0049)	−0.0018(0.0045)	0.0203***(0.0045)	0.0288***(0.0047)	−0.0332***(0.0055)
water	0.0827***(0.0228)	−0.0846***(0.0191)	0.0603***(0.0191)	0.0612***(0.0189)	−0.0753***(0.0242)
lnpergdp	−0.0700(0.0823)	0.1976***(0.0758)	−0.2399***(0.0809)	−0.4010***(0.0763)	0.1644 (0.1130)
secrate	−0.0003(0.0005)	−0.0003(0.0005)	0.0017***(0.0005)	0.0020***(0.0005)	−0.0009 (0.0007)
thirdrate	−0.0005(0.0004)	−0.0001(0.0004)	0.0021***(0.0004)	0.0016***(0.0004)	−0.0006 (0.0006)
urbanization	0.0013(0.0011)	−0.0046***(0.0010)	0.0022**(0.0011)	−0.0002(0.0010)	0.0008(0.0015)
savrate	−0.0003***(0.0001)	0.0003***(0.0001)	−0.0002**(0.0001)	−0.0002**(0.0001)	0.0002(0.0001)
Constant	0.1762(0.7376)	−0.9436(0.6286)	1.7699**(0.7059)	1.8295***(0.6682)	0.0146(0.9288)
City FE	YES	YES	YES	YES	YES
Year FE	YES	YES	YES	YES	YES
cluster	YES	YES	YES	YES	YES
Obs.	47,805	47,805	47,805	47,805	47,805

Standard errors are given in parentheses; ***p < 0.01, **p < 0.05, *p < 0.1.

### 4.3 Heterogeneity analysis

#### 4.3.1 Grouping by region

China has a vast territory, and the level of economic development varies greatly between regions. Therefore, according to the geographical location and the degree of development, we divide China into four regions—eastern, central, western, and northeastern—and investigate the heterogeneous impact of import trade liberalization on the health of residents in the different regions. The regression results in columns (1)–(4) in Table 4 show that the expansion of imports has no significant impact on the health level of people in northeast China and is obviously not beneficial to the health of people in the economically developed eastern region, but can significantly promote the health status of people in the central and western regions. In terms of coefficients, the promotion effect of the west is higher than that of the central region. Thanks to the reform and opening-up policy, unique geographical conditions, and earlier macro policy preference, the eastern region has long been in the first echelon of China’s regional development. But economic growth is often accompanied by serious environmental problems (43). Relevant data show that the pollution level in the eastern region is higher than the national average (44). From the perspective of industrial structure, the secondary industry in the eastern region is on a larger scale, and expanding imports will lead to more industrial production, thereby generating more industrial waste gas and other environmental pollutants, and the emissions of this pollutant are higher than those in the central and western regions (45), which has an extremely bad impact on people’s health. The expansion of the import scale leads to domestic enterprises facing more competition from external products and technology and occupies the development space of the same type of enterprises, which means that these businesses have a stronger sense of survival and pay more attention to the benefits of economic development, so that they may ignore the importance of environmental protection. Therefore, various reasons have led to import trade causing greater harm to the health of people in the eastern region. The biggest difficulty facing the central and western regions, which are restricted by infrastructure and geographical location, is the development dilemma, including backward ideas, single industrial structure, extensive development mode, serious resource dependence, lack of economic development stamina, and so on. Expanding China’s opening-up could effectively stimulate the strong endogenous power and development vitality of the central and western regions. The growing import network will expand the scope of resource selection for production and consumption in the central and western regions, helping to break through the choke point of development, promote the flexible flow of factors, realize the transformation of resource advantages into economic ones, drive the growth of fiscal revenue, improve the public service system including education and health, and ultimately benefit people’s health.

**Table 4:** Heterogeneity analysis—grouping by region and education.

Variables	East	Central	West	Northeast	Highly educated	Low educated
	(1)	(2)	(3)	(4)	(5)	(6)
tariff	5.2391***(1.8359)	−0.8159**(0.3638)	−1.4087**(0.5914)	0.7625(1.0827)	0.9118(2.5092)	1.1948*(0.6148)
age	−0.0145***(0.0014)	−0.0164***(0.0015)	−0.0188***(0.0017)	−0.0177***(0.0019)	−0.0193***(0.0039)	−0.0195***(0.0008)
work	0.2204***(0.0306)	0.1718***(0.0331)	0.0579(0.0370)	0.1671***(0.0411)	0.0978(0.0966)	0.1718***(0.0176)
marri	−0.0368(0.0525)	0.0283(0.0606)	−0.0515(0.0591)	−0.0194(0.0667)	−0.2497**(0.1135)	0.0104(0.0308)
insurance	0.0410(0.0411)	−0.0529(0.0551)	0.0034(0.056)	−0.0542(0.0518)	−0.1930(0.1265)	0.0167(0.0256)
ifsmoke	0.1246***(0.0414)	0.0094(0.0449)	0.0751(0.0483)	−0.0364(0.0514)	−0.0233(0.0930)	0.0413*(0.0237)
ifwine	0.1591***(0.0392)	0.2720***(0.0450)	0.2447***(0.0498)	0.1684***(0.0527)	−0.0178(0.0941)	0.2201***(0.0235)
gender	0.1026**(0.0401)	0.1257***(0.0455)	0.1754***(0.0492)	0.2782***(0.0527)	0.2644***(0.0900)	0.2006***(0.0233)
hukou	−0.0260(0.0426)	−0.0565(0.0534)	0.0429(0.057)	0.0725(0.0606)	−0.0466(0.1159)	−0.0621**(0.0266)
education	0.0242*** (0.0041)	0.0231***(0.0047)	0.0240***(0.0045)	0.0336***(0.0058)	–	–
familysize	0.0247***(0.0085)	0.0049(0.0081)	0.0231**(0.0105)	0.0233(0.0152)	−0.0043(0.0269)	0.0167***(0.0049)
lnfamperinc	0.0296***(0.0075)	0.0244***(0.0090)	0.0251**(0.0102)	0.0277***(0.0103)	0.0039(0.0189)	0.0322***(0.0046)
water	0.1122***(0.0369)	0.0073(0.0356)	0.1138***(0.0364)	0.0629(0.0504)	−0.1346(0.1440)	0.0947***(0.0195)
lnpergdp	−0.1476(0.1945)	−0.7367***(0.2161)	0.3998*(0.2203)	−0.1947(0.2043)	−0.2428(0.3172)	−0.2498***(0.0757)
secrate	−0.0010(0.0011)	−0.0013(0.0011)	−0.0016**(0.0008)	−0.0008(0.0010)	0.0001(0.0021)	−0.0005(0.0005)
thirdrate	−0.0026***(0.0009)	−0.0007(0.0011)	−0.0015*(0.0009)	−0.0016*(0.0009)	−0.0020(0.0018)	−0.0007*(0.0004)
urbanization	0.0086*(0.0046)	0.0004(0.0025)	0.0022*(0.0013)	0.0027(0.0031)	0.0038(0.0059)	0.0021**(0.0010)
savrate	−0.0001(0.0002)	−0.0004**(0.0002)	−0.0001(0.0002)	−0.0010***(0.0002)	0.0000(0.0004)	−0.0004***(0.0001)
Constant	−4.1743***(1.3009)	8.3999***(2.0164)	−2.4056(1.7548)	1.4440(2.0624)	3.0814(2.8168)	1.7271***(0.6560)
City FE	YES	YES	YES	YES	YES	YES
Year FE	YES	YES	YES	YES	YES	YES
cluster	YES	YES	YES	YES	YES	YES
Obs.	15,950	12,335	10,815	8,705	3,176	44,629

Standard errors are given in parentheses; ***p < 0.01, **p < 0.05, *p < 0.1.

#### 4.3.2 Grouping according to education level

To explore the impact of expanding imports on groups with different levels of education, we judge the survey individuals who received tertiary education or above as highly educated, otherwise they are judged as having a low level of education. The regression results are shown in columns (5)–(6) of Table 4. The decline in import tariffs has a negative impact on the health of both those with a high and those with a low education, but it is only significant for the latter groups. This conclusion is consistent with that of Zhang (10). The possible reason for this is that, generally, a higher education level is accompanied by more complete work skills. Good work income provides such people with necessary maintenance capital and also helps them to fight against the employment fluctuation caused by trade liberalization (46, 47). At the same time, the more real and effective information that highly educated people have can help them invest in personal health more reasonably (48). The group with a lower education level lacks both sufficient awareness of a healthy life and enough space and flexibility in career choices. The product substitution effect caused by the expansion of import scale may affect the employment and personal income of this group by affecting enterprise profits. At the same time, the imported inflation caused by imports applies more pressure on the low-income group, limits the range of their daily diet choices, and finally has a negative effect on the health of residents.

#### 4.3.3 Grouping by age

According to the upper limit of China’s legal retirement age (60 years), the sample is divided into an older adults group and a young and middle-aged group. Models (1)–(2) in Table 5 show that trade liberalization has no obvious impact on the health of the older adults, but it has a significant negative impact on the health of the young and middle-aged. In China, people over 60 years of age have basically withdrawn from the labor market, so macroeconomic changes such as unemployment and public education services brought about by the implementation of import trade liberalization policies are unlikely to affect these groups. For those under 60 years of age who are in their own career development stage, the pressures from family and work are greater. An import trade liberalization policy is likely to bring about adjustments in the individual income of residents by acting on the labor market or resulting in occupational mobility. To cope with possible job changes, young and middle-aged people need to invest more time and energy in improving their skills. These factors increase the psychological and physical burden of residents and heighten the risk of suffering from various physical and psychological diseases, such as cancer, anxiety, and depression. In addition, people at this stage of life usually have higher life ideals, which makes them willing to make more of an effort, even at the cost of health, to realize their life goals.

**Table 5:** Heterogeneity analysis—grouping by age, gender, urban and rural.

Variables	Older adults group	Young and middle-aged group	Male	Female	Urban area	Rural area
	(1)	(2)	(3)	(4)	(5)	(6)
tariff	−1.4533(1.4003)	1.3668**(0.6706)	0.4703(0.8520)	1.6850**(0.8256)	−0.1673(0.9947)	3.3530***(1.300)
age	0.0009(0.0032)	−0.0228***(0.0011)	−0.0163***(0.0011)	−0.0170***(0.0012)	−0.0129***(0.0016)	−0.0182***(0.0009)
work	0.3123***(0.0337)	0.1621***(0.0203)	0.1513***(0.0264)	0.1820***(0.0227)	0.2187***(0.0342)	0.1701***(0.0203)
marri	0.0083(0.0466)	0.0341(0.0376)	0.0228(0.0428)	−0.0679(0.0415)	−0.0772(0.0505)	−0.003(0.0361)
insurance	0.0971*(0.0512)	−0.0387(0.0285)	−0.0274(0.0390)	0.0029(0.0325)	−0.0554(0.0390)	0.0006(0.0325)
ifsmoke	0.0932**(0.0387)	0.0455*(0.0275)	0.0683***(0.0249)	−0.0310(0.0619)	−0.0283(0.0410)	0.0799***(0.0275)
ifwine	0.2684***(0.041)	0.1997***(0.0266)	0.2268***(0.025)	0.1385**(0.0585)	0.1543***(0.0415)	0.2371***(0.0270)
gender	0.1087***(0.0411)	0.1606***(0.0271)	–	–	0.2255***(0.0405)	0.1275***(0.0278)
hukou	−0.1139**(0.046)	0.0445(0.0304)	−0.0365(0.0364)	0.0343(0.0369)	–	–
education	0.0225***(0.0042)	0.0269***(0.0027)	0.0226***(0.0033)	0.0278***(0.0033)	0.0273***(0.0042)	0.0244***(0.0028)
familysize	0.0159*(0.0082)	0.0187***(0.0058)	0.0015(0.0067)	0.0319***(0.0070)	0.0092(0.0094)	0.0204***(0.0057)
lnfamperinc	0.0245***(0.0065)	0.0418***(0.0064)	0.0243***(0.0065)	0.0292**(0.0062)	0.0209***(0.007)	0.0309***(0.0059)
water	0.1015***(0.0380)	0.0674***(0.0219)	0.0687**(0.0280)	0.0791***(0.0264)	0.0505(0.0528)	0.0832***(0.0209)
lnpergdp	−0.1380(0.1608)	−0.2893***(0.0891)	−0.2148*(0.1114)	−0.2767***(0.0990)	0.0870(0.1347)	−0.4055***(0.0953)
secrate	−0.0002(0.0010)	−0.0006(0.0005)	−0.0008(0.0007)	−0.0002(0.0006)	−0.0019**(0.0009)	0.0006(0.0006)
thirdrate	−0.0004(0.0008)	−0.0008*(0.0005)	−0.0009(0.0006)	−0.0007(0.0005)	−0.0016**(0.0007)	−0.0001(0.0005)
urbanization	0.0022(0.0023)	0.0022**(0.0011)	0.0040***(0.0014)	0.0003(0.0014)	0.0020(0.0026)	0.0031***(0.0011)
savrate	−0.0003(0.0002)	−0.0004***(0.0001)	−0.0004***(0.0001)	−0.0004***(0.0001)	−0.0001(0.0002)	−0.0004***(0.0001)
Constant	1.8293(1.4527)	1.8143**(0.7314)	2.0419**(0.9294)	1.3456(0.8852)	−0.6794(1.0900)	0.8013(1.2003)
City FE	Yes	Yes	Yes	Yes	Yes	Yes
Year FE	Yes	Yes	Yes	Yes	Yes	Yes
cluster	Yes	Yes	Yes	Yes	Yes	Yes
Obs.	11,957	35,848	22,927	24,878	14,014	33,791

Standard errors are given in parentheses; ***p < 0.01, **p < 0.05, *p < 0.1.

#### 4.3.4 Grouping by gender

Models (3)–(4) in Table 5 show that, in the regression results grouped by gender, import trade liberalization is not conducive to either men’s or women’s health, but has a significant negative impact on women’s health. Generally speaking, the skill level of most of the female labor force in developing countries is lower than that of the male labor force (49), and women spend more energy on childbearing and raising offspring, so they are usually in a relatively disadvantageous position in the labor market. Expanding imports will lead to significant adjustments in the gender structure of employment in enterprises, resulting in a decrease in the number of female employees. This downward trend is more pronounced in industries with a larger female workforce, labor-intensive industries, and developing countries (50). Most studies have shown that trade liberalization aggravates gender employment discrimination in general (51), and the development of technology (such as investment in machinery and equipment) will produce a crowding out effect on the female labor force (52) and widen the gender wage gap (53). In addition, in the face of the price increases caused by imports, women from low-income families may extend their working hours out of a sense of responsibility for changes in the family welfare so as to alleviate the economic burden on the whole family. All of these factors will make it more difficult for women to take care of their family and work. In the long run, women’s bodies and minds will be destroyed.

#### 4.3.5 Grouping by urban and rural areas

Models (5)–(6) in Table 5 show the regression results for urban and rural samples. Tariff reduction will significantly reduce the health level of the rural population and improve the health status of the urban population, but the positive effect is not significant. The reason for the heterogeneity between urban and rural areas may be that the rural population is limited by education and skill levels, and mostly engages in low-tech jobs. Lowering import tariffs has a greater impact on the employment and wages of low-skilled workers, and the higher the degree of imports, the greater the negative impact (54), which hinders rural residents’ intake of healthy and nutritious food. In addition, the import of unhealthy food may introduce unhealthy eating habits (3), while the rural population may develop unhealthy lifestyles due to their limited level of knowledge and the lack of sufficient ability in terms of discrimination and self-control. Urban residents often occupy medium- and high-skilled jobs by virtue of a relatively good cultural education, and have a strong adaptability to foreign competition, so their own health will not be negatively affected as much.

### 4.4 Mechanism analysis

#### 4.4.1 Environmental mechanism

Regional environmental conditions involve air, water, and other aspects. To test the real role of the environmental mechanism, this paper introduces four variables to measure environmental quality: (1) green coverage; (2) annual average value of PM2.5; (3) industrial wastewater discharge; and (4) industrial waste gas discharge. The data are from the EPS statistical database and the International Geoscience Information Network Center of Columbia University. Because Columbia University has not released the haze data for all cities in 2018, the environmental mechanism test using PM2.5 data is conducted for the period 2010–2016. At the same time, some cities with incomplete industrial wastewater and waste gas data in the sample period are deleted. Columns (1)–(4) of Table 6 report the regression results after introducing the interaction between tariff and the four environmental mechanism variables. From the significance of the interaction, we can see that import trade liberalization has an impact on health through the environmental mechanism. More specifically, in the areas with severe haze and heavy industrial wastewater and gas emissions—that is, the areas with serious pollution—the increase in import trade has a significant negative impact on health by aggravating the degree of pollution. Expanding green coverage is an effective way to reduce the health hazards of trade liberalization.

**Table 6:** Mechanism test.

Variables	Green coverage	PM2.5	Industrial Wastewater	Industrial Waste gas	Middle school per 10,000 people	Medical institutions per 10,000 people	Medical technicians per 10,000 people	Income gap -Ratio	Income gap -Difference
	(1)	(2)	(3)	(4)	(5)	(6)	(7)	(8)	(9)
tariff	1.3758**(0.6816)	0.8441 (0.9347)	−4.5735**(1.8791)	−4.6233**(1.8974)	2.3847**(1.0242)	1.3711**(0.6033)	1.4054**(0.6146)	1.7510*(0.9435)	2.1707***(0.8038)
tariff*environment/tariff*pubservice/tariff*gap	−0.0013*(0.0007)	0.0032**(0.0015)	0.0008*(0.0004)	0.0012***(0.0004)	−0.0048**(0.0022)	−0.0006*(0.0003)	−0.0006*(0.0003)	−0.1722(0.1594)	0.0000(0.0000)
environment/pubservice/gap	0.0012*(0.0007)	−0.0034***(0.0013)	−0.0006(0.0004)	−0.0012***(0.0004)	0.0037**(0.0019)	0.0007**(0.0003)	0.0006*(0.0003)	0.1343(0.1403)	0.0000(0.0000)
age	−0.0166***(0.0008)	−0.0182***(0.0008)	−0.0172***(0.0009)	−0.0172***(0.0009)	−0.0167***(0.0008)	−0.0167***(0.0008)	−0.0168***(0.0008)	−0.0167***(0.0008)	−0.0167***(0.0008)
work	0.1682***(0.0176)	0.1450***(0.0183)	0.1620***(0.0187)	0.1622***(0.0188)	0.1669***(0.0172)	0.1659***(0.0172)	0.1657***(0.0172)	0.1667***(0.0172)	0.1658***(0.0172)
marri	−0.0238(0.0302)	−0.0071(0.0316)	−0.0137(0.0319)	−0.0142(0.0319)	−0.0215(0.0295)	−0.0216(0.0295)	−0.0282(0.0296)	−0.0220(0.0295)	−0.0221(0.0295)
insurance	0.0010(0.0258)	−0.0389(0.0269)	−0.0006(0.0271)	0.0020(0.0271)	−0.0084(0.0249)	−0.0091(0.0249)	−0.0059(0.0250)	−0.0093(0.0250)	−0.0085(0.0249)
ifsmoke	0.0452*(0.0236)	0.0518**(0.0242)	0.0510**(0.0254)	0.0510**(0.0254)	0.0483**(0.0230)	0.0482**(0.0230)	0.0499**(0.0230)	0.0483**(0.0230)	0.0479**(0.0230)
ifwine	0.2154***(0.0233)	0.2059***(0.0245)	0.2091***(0.0250)	0.2087***(0.0250)	0.2097***(0.0229)	0.2105***(0.0228)	0.2077***(0.0229)	0.2103***(0.0228)	0.2105***(0.0228)
gender	0.1586***(0.0236)	0.1700***(0.0241)	0.1646***(0.0252)	0.1647***(0.0252)	0.1588***(0.0230)	0.1588***(0.0230)	0.1574***(0.0231)	0.1587***(0.0230)	0.1590***(0.0230)
hukou	−0.0257(0.0267)	0.0241(0.0274)	0.0103(0.0281)	0.0109(0.0281)	−0.0029(0.0258)	−0.0020(0.0258)	−0.0019(0.0259)	−0.0022(0.0258)	−0.0030(0.0258)
education	0.0245***(0.0024)	0.0245***(0.0024)	0.0231***(0.0025)	0.0231***(0.0025)	0.0250***(0.0023)	0.0250***(0.0023)	0.0252***(0.0023)	0.0250***(0.0023)	0.0249***(0.0023)
familysize	0.0170***(0.0049)	0.0183***(0.0052)	0.0176***(0.0052)	0.0177***(0.0052)	0.0173***(0.0049)	0.0172***(0.0049)	0.0173***(0.0049)	0.0172***(0.0049)	0.0173***(0.0049)
lnfamperinc	0.0271***(0.0046)	0.0536***(0.0072)	0.0285***(0.0050)	0.0287***(0.0050)	0.0267***(0.0045)	0.0269***(0.0045)	0.0271***(0.0045)	0.0268***(0.0045)	0.0266***(0.0045)
water	0.0780*** (0.0197)	0.0627***(0.0205)	0.0975***(0.0211)	0.0977***(0.0211)	0.0723***(0.0192)	0.0732***(0.0192)	0.0735***(0.0192)	0.0728***(0.0192)	0.0726***(0.0192)
lnpergdp	−0.2593***(0.0745)	−0.4936***(0.0979)	−0.1422***(0.0551)	−0.1648***(0.0566)	−0.2885***(0.0827)	−0.2442***(0.0734)	−0.2279***(0.0740)	−0.2836***(0.0769)	−0.2647***(0.0739)
secrate	−0.0003 (0.0005)	0.0011*(0.0006)	−0.0016***(0.0005)	−0.0015***(0.0005)	−0.0005(0.0005)	−0.0005(0.0005)	−0.0006(0.0005)	−0.0005(0.0005)	−0.0006(0.0005)
thirdrate	−0.0008**(0.0004)	0.0004 (0.0005)	−0.0014***(0.0005)	−0.0013***(0.0005)	−0.0009**(0.0004)	−0.0007*(0.0004)	−0.0010**(0.0004)	−0.0008**(0.0004)	−0.0011***(0.0004)
urbanization	0.0025***(0.001)	0.0040***(0.0011)	0.0014(0.0011)	0.0012(0.0011)	0.0017*(0.0010)	0.0017*(0.0010)	0.0023**(0.0010)	0.0018 (0.0011)	0.0024**(0.0010)
savrate	−0.0004***(0.0001)	−0.0005***(0.0001)	−0.0003***(0.0001)	−0.0004***(0.0001)	−0.0004***(0.0001)	−0.0004***(0.0001)	−0.0004***(0.0001)	−0.0004***(0.0001)	−0.0005***(0.0001)
Constant	1.4590**(0.6945)	3.9317***(0.8598)	6.6619***(2.2719)	6.9965***(2.3621)	1.0381(0.7066)	1.2940**(0.6449)	1.1231(0.7015)	1.4659**(0.7454)	0.9993(0.7019)
City FE	Yes	Yes	Yes	Yes	Yes	Yes	Yes	Yes	Yes
Year FE	Yes	Yes	Yes	Yes	Yes	Yes	Yes	Yes	Yes
cluster	Yes	Yes	Yes	Yes	Yes	Yes	Yes	Yes	Yes
Obs.	45,705	37,920	40,140	40,140	47,805	47,805	47,630	47,805	47,805

Standard errors are given in parentheses; ***p < 0.01, **p < 0.05, *p < 0.1.

#### 4.4.2 Public service mechanism

The positive effect of education on health has been widely confirmed. Receiving an education will increase people’s safety awareness and make them more inclined to choose a healthy lifestyle (55). The employment improvement generated by education capital can affect the budget constraint set for health investment through income (56). At the same time, medical services can also provide health protection for residents through the prevention and treatment of diseases. To this end, we use three indicators to measure the quality of public services in various cities, including the number of secondary schools per 10,000 people, medical institutions per 10,000 people, and medical technicians per 10,000 people, to verify the mechanism of public service supply. Models (5)–(7) in Table 6 report the regression results of the mechanism with these three indicators. The interaction terms between import tariffs and education and medical services are significantly negative, which means that the adverse impact of import trade liberalization on health will be weakened in areas where high-quality public services are provided. This confirms the mechanism of public services in the health effect of import trade, and shows that high-quality education and medical services are strong weapons to help residents fight against the negative impact of trade liberalization.

#### 4.4.3 Income gap mechanism

As mentioned above, the impact of the income gap on residents’ health may have two opposite effects. On the one hand, the aggravation of income inequality will deprive the legitimate health needs of the low-income class by inducing social instability, aggravating the psychological imbalance of the group, strengthening the polarization of public service demand, and tightening the health investment credit constraints. On the other hand, the technological innovation generated by the demand for high-quality public services and the resulting technology spillover and government tax revenue increase may also objectively improve the existing supply of public services and benefit other groups, except the high-income groups. The impact of the income gap on health is thus still uncertain. This paper uses the ratio and difference of per capita disposable income of urban and rural residents as the alternative variables for the income gap to verify the mechanism. In models (8)–(9) of Table 6, the interaction terms between import tariff and income gap are not significant, indicating that import trade liberalization does not affect residents’ health through income inequality. This conclusion is consistent with the research results of Dollar et al. (57).

## 5 Conclusion and enlightenment

This paper is based on the CFPS data from the China Institute of Social Science Survey, Peking University, using an IVprobit model to investigate the impact of import trade liberalization on residents’ health. From the perspective of the environment, public services, and the income gap, this paper explores the mechanisms for the effect of import expansion on health and classifies the survey samples according to region, education, age, gender, and urban–rural standards. We test the robustness of the benchmark regression results by replacing instrumental variables, changing empirical methods, and changing health measurement indicators. The results show that, on the whole, the import expansion caused by tariff reduction is not conducive to the health of residents, and a variety of robustness tests support this view. Environmental pollution is a significant intermediary mechanism of this negative effect, and increasing green coverage and improving public education and public health services can help alleviate the negative impact, while the income gap is not significant in the mechanism test. Heterogeneity analysis shows that import trade liberalization is obviously detrimental to the health of people in eastern regions, people with low education levels, people under 60 years of age, women, and people living in rural areas. The conclusions of this paper provide rich and useful policy inspiration:

(1) Environmental protection is closely related to people’s health. China should always adhere to the important concept that clear waters and lush mountains are invaluable assets, attach great importance to the protection of the ecological environment in the process of development, and prevent enterprises from blindly pursuing short-term economic benefits and ignoring environmental consequences in face of import competition pressure. We need to encourage technological innovation and R&D; make rational use of the import spillover effect to realize the absorption, imitation, and learning of high-end technologies in imported products; and reduce corporate pollutant emissions from the source.

(2) Improving the public service system is a powerful measure by which to combat the negative health effect of import trade. Therefore, China should ensure the development of basic public education and medical systems. The government should vigorously promote high-quality education, optimize the allocation of educational resources, gradually narrow the educational gap between urban and rural areas and eastern and western regions, increase educational subsidies to specific areas and groups (e.g., those in remote and poverty-stricken areas, ethnic minority areas, and students with financial difficulties), and promote the equalization of public education services. China should also establish a multi-level medical security system, strengthen the construction of primary medical and health institutions, reduce the difference in medical and health resources between different regions through policy preference. At the same time, China should therefore popularize knowledge of mental health at the national level and strengthen a self-management consciousness regarding mental health.

(3) The results of the heterogeneity analysis indicate that the health hazards of import trade liberalization differ by group. This paper argues that the health of those with a lower education, the rural population suffering from limitations, the eastern population facing greater external environmental pressure, young and middle-aged people, and women who need more consideration for career development and family life will be more greatly affected by import liberalization. Based on this, the government should adopt a problem-oriented strategy and provide psychological counseling for such people. China should optimize the social environment through macro policies, including the creation of employment opportunities, alleviating gender discrimination, solving important practical problems related to the national economy and people’s livelihood, and creating a good and appropriate social development environment for all residents.

## Data Availability

The original contributions presented in the study are included in the article/supplementary material, further inquiries can be directed to the corresponding author.

## Footnotes

^1^As we know, the COVID-19 that broke out in 2019 has greatly affected the import policies of countries and the health level of people in the following years. Therefore, we limit the research period to the year before this special event.

^2^Minors under the age of 16 are not the focus of this study.

^3^In the CFPS survey itself, the regions of interest did not include Inner Mongolia, Xinjiang, Qinghai, Hainan,Ningxia, Tibet, Taiwan, Hong Kong, and Macao. Guizhou was excluded in the follow-up empirical analysis due to the serious lack of variables.

## References

[1] Owen AL Wu S. Is trade good for your health? Rev Int Econ. (2007) 15:660–82. doi: 10.1111/j.1467-9396.2007.00677.x

[2] Colantone I Crinò R Ogliari L. The hidden cost of globalization, import competition and mental distress. SSRN Working Paper. (2015). doi: 10.2139/ssrn.2694447

[3] Giuntella O Rieger M Rotunno L. Weight gains from trade in foods, evidence from Mexico. NBER Working Paper. (2018) 122:103277. doi: 10.1016/j.jinteco.2019.103277

[4] Atkin D. Trade, tastes and nutrition in India. Am Econ Rev. (2012) 103:1629–63. doi: 10.1257/aer.103.5.1629

[5] Lang M Mcmanus TC Schaur G. The effects of import competition on health in the local economy. Working Paper. (2016) 28:44–56. doi: 10.1002/hec.3826, PMID: 30230125

[6] Mcmanus TC Schaur G. The effects of import competition on worker health. J Int Econ. (2016) 102:160–72. doi: 10.1016/j.jinteco.2016.06.003 30230125

[7] Liu KH Tong JD Liu RJ. Health costs of China’s export expansion, evidence from adult incidence rate. Chin Ind Econ. (2019) 8:118–35. doi: 10.19581/j.cnki.ciejournal.2019.08.007

[8] Feng J Xie Q Zhang XH. Trade liberalization and the health of working-age adults, evidence from China. World Dev. (2021) 139:105344. doi: 10.1016/j.worlddev.2020.105344

[9] Fan HC Lin FQ Lin S. The hidden cost of trade liberalization, input tariff shocks and worker health in China. J Int Econ. (2020) 126:1–24. doi: 10.1016/j.jinteco.2020.103349

[10] Zhang MA. How does trade liberalization affect people’s health?—Evidence from China’s accession into WTO? Chin Econ Q. (2021) 21:819–42. doi: 10.13821/j.cnki.ceq.2021.03.04

[11] Lei QY Qi CJ Sun CR. Will import trade liberalisation improve the health of Chinese residents—research based on CGSS data from 2010 to 2015. J Int Trade. (2021) 9:51–69. doi: 10.13510/j.cnki.jit.2021.09.004

[12] Grossman M. On the concept of health capital and the demand for health. J Polit Econ. (1972) 80:223–55. doi: 10.1086/259880

[13] Chen Y Ebenstein A Greenstone M Li HB. Evidence on the impact of sustained exposure to air pollution on life expectancy from China’s Huai river policy. Proc Natl Acad Sci USA. (2013) 110:12936–41. doi: 10.1073/pnas.1300018110, PMID: 23836630 PMC3740827

[14] Lovely ME Popp D. Trade, technology, and the environment, why have poor countries regulated sooner? NBER Working Papers. (2008)

[15] Guo SL. Research on enterprises intermediate input imports and the effect of pollution emission. World Econ Stud. (2019) 9:67–77+135. doi: 10.13516/j.cnki.wes.2019.09.005

[16] Managi S Hibiki A Tsurumi T. Does trade openness improve environmental quality? J Environ Econ Manag. (2009) 58:346–63. doi: 10.1016/j.jeem.2009.04.008

[17] Cole MA. Trade, the pollution haven hypothesis and the environmental Kuznets curve. Examin Link Ecol Econ. (2004) 48:71–81. doi: 10.1016/j.ecolecon.2003.09.007

[18] He LY Huang G. Are China’s trade interests overestimated? Evidence from firms’ importing behavior and pollution emissions. Chin Econ Rev. (2022) 71:101738–18. doi: 10.1016/j.chieco.2021.101738

[19] Greenstone M Hanna R. Environmental regulations, air and water pollution, and infant mortality in India. Am Econ Rev. (2011) 104:3038–72. doi: 10.2139/ssrn.1907924

[20] Liao QM Wu JW. Analysis on the influence of comprehensive medical and health service level on health output. Chin Health Econ. (2016) 12:58–9. doi: 10.7664/CHE20161216

[21] Huang F Cheng Q. From universal coverage to equal coverage: equity analysis of national health security. Financ Trad Res. (2024) 1:71–83. doi: 10.19337/j.cnki.34-1093/f.2024.01.006

[22] Rodric D. Why do more open economies have bigger governments? CEPR Discussion Papers. (1998)

[23] Wang YS Cheng H. Does export rebates promote local fiscal revenue? Based on urban panel data in China from 2004 to 2011. Fin Trade Res. (2019) 4:56–70. doi: 10.19337/j.cnki.34-1093/f.2019.04.005

[24] Zhou LA Chen W. Local fiscal burden and public good provision, impact of rural tax-for-fee reform. Chin Econ Q. (2015) 2:417–34. doi: 10.13821/j.cnki.ceq.2015.02.001

[25] Dai M Zhang YF Huang W. How does trade liberalization affect China’s regional labor market? Manag World. (2019) 6:56–69. doi: 10.19744/j.cnki.11-1235/f.2019.0079

[26] Hu C Ji T Chen YB. Trade liberalization and informal employment. Nankai Econ Stud. (2019) 12:3–24. doi: 10.14116/j.nkes.2019.02.001

[27] Subramanian SV Kawachi I. The association between state income inequality and worse health is not confounded by race. Int J Epidemiol. (2003) 32:1022–8. doi: 10.1093/ije/dyg245 14681268

[28] Wang HM Wang YQ Xu RZ. Effect of income and income inequality on rural residents’ health, based on shapley value decomposition. J Nanjing Agr Univ. (2014) 2:28–34.

[29] Wei QG Shang QQ. Beyond income inequality: a novel dissecting of the relationship between happiness inequality and residents’ health. Acad Monthly. (2024) 56:136–48. doi: 10.19862/j.cnki.xsyk.000802

[30] Kennedy BP Kawachi I Prothrowstith D Lochner K Gupta V. Social capital, income inequality, and firearm violent crime. Soc Sci Med. (1998) 47:7–17. doi: 10.1016/S0277-9536(98)00097-5, PMID: 9683374

[31] Mayer SE Sarin A. Some mechanisms linking economic inequality and infant mortality. Soc Sci Med. (2005) 60:439–55. doi: 10.1016/j.socscimed.2004.06.005, PMID: 15550294

[32] Krugman P. The spiral of inequality. Mother Jones. (1996) 21:44–9.

[33] Yu YY Feng J. Review on the relationship between income gap and health. Econ Perspect. (2006) 7:92–6.

[34] Wennemo I. Infant mortality, public policy and inequality—a comparison of 18 industrialised countries 1950-1985. Sociol Health Ill. (1993) 15:429–46. doi: 10.1111/j.1467-9566.1993.tb00354.x

[35] Judge K Patterson I. Poverty, income inequality and health. Working Paper. (2001)

[36] Li Q Zhao R Zhang TL. How does becoming widowed affect the rural elderly’s health? Based on the data of CHARLS. Nankai Econ Stud. (2022) 2:157–76. doi: 10.14116/j.nkes.2022.02.010

[37] Ma QS Chen MC Hu F. The health impact of healthy city initiatives for the elderly: evidence from the CHARLS data. Chin J Pop Sci. (2023) 37:114–28.

[38] Ni CX Shao BK Cong ZL Wang Z. Impact of age-friendly community renovations on the elderly’s health. Chin J Pop Sci. (2024) 38:113–28.

[39] Finkelstein A Tanbman S Wright B Bernstein M Gruber J Allen H. The Oregon health insurance experiment, evidence from the first year. Q J Econ. (2012) 127:1057–106. doi: 10.1093/qje/qjs020 23293397 PMC3535298

[40] Cutler DM Zeckhauser RJ. The anatomy of health insurance. Hand Health Econ. (2000) 1:563–643. doi: 10.1016/S1574-0064(00)80170-5

[41] Angrist JD Pischke J. Mostly harmless econometrics, an empiricists companion. Princeton: Princeton University Press (2009).

[42] Ma S Lai MT. Exogenous increase of labor cost and FDI entry: from the perspective of minimum wage. Chin Ind Econ. (2020) 6:81–99. doi: 10.19581/j.cnki.ciejournal.2020.06.005

[43] Pata UK. Do renewable energy and health expenditures improve load capacity factor in the USA and Japan? A new approach to environmental issues. Eur J Health Econ. (2021) 22:1427–39. doi: 10.1007/s10198-021-01321-0, PMID: 34019219

[44] Ban L Yuan XL He B. Regional differences and emission reduction paths to China’s environmental pollution. J Xi’an Jiaotong Univ. (2018) 3:34–43. doi: 10.15896/j.xjtuskxb.201803004

[45] Shao J Shi ZK Zhu JM. Import and the green transformation development of Chinese cities—a study based on green total factor productivity. J Int Trade. (2020) 12:51–64. doi: 10.13510/j.cnki.jit.2020.12.004

[46] Kemeny T Rigby D Cooke A. Cheap imports and the loss of US manufacturing jobs. World Econ. (2013) 38:1555–73. doi: 10.1111/twec.12238

[47] Greenland A Lopresti J. Import exposure and human capital adjustment: evidence from the US. J Int Econ. (2016) 100:50–60. doi: 10.2139/ssrn.2262919

[48] Kenkel DS. Health behavior, health knowledge, and schooling. J Polit Econ. (1991) 99:287–305. doi: 10.1086/261751

[49] Zhang XW Xi YL. Trade liberalization and gender discrimination, literature review. Econ Perspect. (2012) 12:136–43.

[50] Li D Chen J Sun CR. Import trade liberalization and gender structure of firm employment. World Econ Papers. (2023) 6:20–36.

[51] Fan NN Li RL. Trade liberalization and firms’ gender employment gap, based on China’s micro-level enterprises database. Intl Econ Trade Res. (2017) 9:54–69. doi: 10.13687/j.cnki.gjjmts.2017.09.005

[52] Ozler S. Export led industrialization and gender differences in job creation and destruction, micro-evidence from Turkish manufacturing sector. Working Paper. (2001).

[53] Seguino S. The effects of structural change and economic liberalization on gender wage differentials in South Korea and Taiwan. Camb J Econ. (2000) 24:437–59. doi: 10.1093/cje/24.4.437

[54] Luo J Chen JG. Trade in intermediate products, technological progress and labor employment in manufacturing industry. Asia-pac Econ Rev. (2014) 6:49–58. doi: 10.16407/j.cnki.1000-6052.2014.06.004

[55] Cutler DM Lleras-Muney A. Understanding differences in health behaviors by education. J Health Econ. (2010) 29:1–28. doi: 10.1016/j.jhealeco.2009.10.003, PMID: 19963292 PMC2824018

[56] Moen ER. Education, ranking and competition for jobs. J Labor Econ. (1999) 17:694–723. doi: 10.1086/209936

[57] Dollar D Kleineberg T Kraay A. Growth still is good for the poor. Eur Econ Rev. (2016) 81:68–85. doi: 10.1016/j.euroecorev.2015.05.008

